# Optimizing Treatment of Parkinson’s Disease

**DOI:** 10.3390/jpm12020245

**Published:** 2022-02-09

**Authors:** Dag Nyholm, Filip Bergquist

**Affiliations:** 1 Department of Medical Sciences, Neurology at Uppsala University, 75185 Uppsala, Sweden; 2 Department of Pharmacology, Institute of Neuroscience and Physiology, University of Gothenburg, 41296 Gothenburg, Sweden

The holy grail of therapy in Parkinson’s disease (PD) is treatment that would halt the disease process or restore the degenerated neuronal circuits. Despite the many advances in stem cell technology, the genetic characterization of disease and a better understanding of the molecular processes involved in PD pathology, this shift in treatment strategies is still in the future. However, while the work towards a cure for PD continues, improvements in current symptomatic treatments are having an immediate (or soon to come) impact on disease burden for patients with PD and their caregivers. This Special Issue is dedicated to the optimization of PD treatment and contains several examples of the individualization of treatments with standard PD drugs based on novel sensor and information technology [1,2,3], as well as studies of factors important for making individualized decisions for advanced treatment with continuous dopaminergic drug infusion and deep brain stimulation, DBS [1,4,5,6]. Several symptoms of PD can be difficult to treat and both the review of gastrointestinal dysfunction and the systematic review of the effect of foot orthoses and shoes in PD address some of those areas [7,8]. 

Optimizing the treatment of dopaminergic drugs is important for balancing efficacy and adverse effects. The results from intestinal levodopa infusion trials demonstrate that stable plasma concentrations as well as personalized drug levels are the determining factors for efficacy and tolerability. The article by Öthman et al. is the first report on the clinical use of the most recently marketed infusion therapy for PD in Europe: the levodopa/entacapone/carbidopa intestinal gel [6]. Some of the patients switched from previous levodopa/cabidopa infusion and perceived the smaller pump size as an important improvement.

At earlier stages of disease, infusion treatments may be too invasive, so non-invasive treatment alternatives that aim at continuous dopaminergic stimulation are welcome. One such example is the ongoing development of a mucoadhesive film of the dopamine agonist ropinirole, intended for buccal administration, as presented by Di Prima et al. [9]. Another example, using the oral route of administration, is the dispersible levodopa/carbidopa microtablet formulation, which is approved in a few European countries, and enables dose adjustments in steps of 5 mg of levodopa. The report by Grétarsdóttir et al. shows that PD patients may find this treatment efficacious and user-friendly [2].

One of the major problems with the oral administration of levodopa in PD patients in the fluctuation phase is the erratic gastric emptying, which is why infusion therapies have been developed. In the review by Han et al., it is reported that 70–90% of PD patients have gastroparesis [8]. The current resurgence of the interest in understanding the role of the gastrointestinal tract in PD will likely result in new additional treatments for PD in the near future. 

The development of affordable wearable motion sensors of the type used in smartphones has resulted in several devices with regulatory approval for evaluating and monitoring PD, but the clinical impact of their use is still unclear. Sundgren and coworkers report that neurologists at a specialized movement disorder unit found novel information that altered the management in more than a third of patients with PD after monitoring with a commercially available device [3], and Kilinçalp et al. show that results from the same device have predictive value for the success of levodopa/carbidopa intestinal infusion in PD patients with handicapping symptom fluctuations [1]. These two studies illustrate that supplementing patient evaluation with objective measurements can further the treatment effects of both standard and advanced therapy. 

Sensors can also be used for other movement disorders, such as essential tremor. Kim et al. report promising results on non-invasive electrical stimulation of peripheral nerves, as a potential treatment for tremor reduction [10]. The method may be particularly suitable for patients who are not eligible for DBS surgery or who have insufficient tremor reduction from DBS.

Factors important for the outcome of continuous apomorphine infusion therapy are described by Henriksen and Staines in one of the largest retrospective studies on the real-life use of apomorphine infusion, and several suggestions for increasing the chance of long-term efficacy are made [4]. 

When the outcome of one advanced treatment is insufficient, it is often possible to try another, but sometimes combinations of two advanced treatments are considered. van Poppelen et al. provide a single-center case series and a systematic review suggesting that a change of therapy, including add-on advanced treatment, often results in improved outcome when the first treatment is insufficient [5]. Interestingly, such examples are given also by other authors in this issue [2,6].

The systematic review by Reina-Bueno et al. highlights the need for non-pharmaceutical devices for optimizing treatment of PD, here exemplified by shoes and orthoses for lower-limb problems [7]. Considering that balance problems may lead to falls and fractures, research on footwear for improving postural stability is an important contribution. 

Altogether, this Special Issue highlights that the treatment of Parkinson’s disease is not just symptomatic; it is effectively symptomatic treatment but with important gaps that remain to be filled. The different contributions also show the significant potential for better individualized treatment optimizations that could be achieved regardless of when and if there is a cure for the disease.

## Data Availability

Not applicable.

## References

[1] Kilinçalp G., Sjöström A.-C., Eriksson B., Holmberg B., Constantinescu R., Bergquist F. (2022). Predictive value of ambulatory objective movement measurement for outcomes of levodopa/carbidopa intestinal gel infusion. J. Pers. Med..

[2] Grétarsdóttir H.M., Widman E., Johansson A., Nyholm D. (2021). Personalized Medicine Approach in Treating Parkinson’s Disease, Using Oral Administration of Levodopa/Carbidopa Microtablets in Clinical Practice. J. Pers. Med..

[3] Sundgren M., Andréasson M., Svenningsson P., Noori R.-M., Johansson A. (2021). Does Information from the Parkinson KinetiGraphTM (PKG) Influence the Neurologist’s Treatment Decisions?—An Observational Study in Routine Clinical Care of People with Parkinson’s Disease. J. Pers. Med..

[4] Henriksen T., Staines H. (2021). Continuous Subcutaneous Apomorphine Infusion in Parkinson’s Disease: A Single-Center, Long-Term Follow-Up Study of the Causes for Discontinuation. J. Pers. Med..

[5] Van Poppelen D., Tromp A.N.M., de Bie R.M.A., Dijk J.M. (2021). Combined and Sequential Treatment with Deep Brain Stimulation and Continuous Intrajejunal Levodopa Infusion for Parkinson’s Disease. J. Pers. Med..

[6] Öthman M., Widman E., Nygren I., Nyholm D. (2021). Initial Experience of the Levodopa–Entacapone–Carbidopa Intestinal Gel in Clinical Practice. J. Pers. Med..

[7] Reina-Bueno M., Calvo-Lobo C., López-López D., Palomo-López P., Becerro-De-Bengoa-Vallejo R., Losa-Iglesias M.E., Romero-Morales C., Navarro-Flores E. (2021). Effect of Foot Orthoses and Shoes in Parkinson’s Disease Patients: A PRISMA Systematic Review. J. Pers. Med..

[8] Han M.N., Finkelstein D.I., McQuade R.M., Diwakarla S. (2022). Gastrointestinal Dysfunction in Parkinson’s Disease: Current and Potential Therapeutics. J. Pers. Med..

[9] Di Prima G., Campisi G., De Caro V. (2020). Amorphous Ropinirole-Loaded Mucoadhesive Buccal Film: A Potential Patient-Friendly Tool to Improve Drug Pharmacokinetic Profile and Effectiveness. J. Pers. Med..

[10] Kim J., Wichmann T., Inan O.T., DeWeerth S.P. (2022). Analyzing the Effects of Parameters for Tremor Modulation via Phase-Locked Electrical Stimulation on a Peripheral Nerve. J. Pers. Med..

